# Anterolateral and accessory anterolateral portals are safe to avoid subcutaneous nerve injury during subtalar arthroscopy—Definition of safe zones for standard lateral portals

**DOI:** 10.1002/ksa.12463

**Published:** 2024-09-29

**Authors:** Lena Hirtler, Vinzenz Bussek, Markus Kleinberger, Madeleine Willegger

**Affiliations:** ^1^ Division of Anatomy, Center for Anatomy and Cell Biology Medical University of Vienna Vienna Austria; ^2^ Department of Orthopedics and Trauma Surgery Medical University of Vienna Vienna Austria

**Keywords:** peroneal nerve, safe zones, subcutaneous nerve injury, subtalar arthroscopy, sural nerve

## Abstract

**Purpose:**

Injury to the superficial peroneal nerve (SPN) or the sural nerve (SN) is a common complication in subtalar arthroscopy. The purpose of this anatomical study was to evaluate the distance to surrounding subcutaneous nerves in the vicinity of three standard arthroscopic portals for subtalar joint arthroscopy and through actual portal placement for arthroscopic procedures, in order to define anatomical safe zones.

**Methods:**

Forty paired fresh‐frozen foot‐and‐ankle specimens were used. Subtalar arthroscopy using a three‐portal technique (anterolateral [AL], posterolateral [PL] and accessory anterolateral [AAL] portals) was performed. After completion of subtalar arthroscopy, the portals were marked, and all surrounding subcutaneous nerves, that is, the branches of the SPN and SN, were dissected. The distance of the nearest nerve at the level of the respective portal was measured and potential injury was recorded.

**Results:**

The nearest nerve at the level of the AL portal was the intermediate dorsal cutaneous nerve at a mean of 15.4 ± 5.1 mm medial to the portal. The nearest nerve at the level of the AAL portal was the lateral dorsal cutaneous nerve at a mean of 17.7 ± 4.8 mm, being lateral to the portal. The nearest nerve at the level of the PL portal was the SN at a mean of 6.7 ± 4.7 mm anterior to the portal. Based on the measurements, safe zones were defined.

**Conclusions:**

Placement of the AL and AAL portals in subtalar arthroscopy is saved using standard anatomical landmarks and a thorough surgical technique. At the level of the PL portal, the SN is the most endangered structure in subtalar arthroscopy. Surgeons should be aware of the proximity of the SN to the PL portal and take the utmost care during portal placement and instrument insertion to avoid iatrogenic injury. The risk of nerve damage during portal placement may be reduced when positioning the portals in the defined safe zones.

**Level of Evidence:**

Not applicable.

**Abbreviations**
AAL: accessory anterolateral
AL: anterolateral
IDCN: intermediate dorsal cutaneous nerve
LDCN: lateral dorsal cutaneous nerve
MDCN: medial dorsal cutaneous nerve
PL: posterolateral
SN: sural nerve
SPN: superficial peroneal nerve

## INTRODUCTION

Subtalar arthroscopy was first described by Parisien in 1985 [37]. Through continuous technical development it has evolved from a niche technology to a versatile surgical tool. Indications for subtalar arthroscopy range from simple joint debridement to more complex procedures such as arthroscopic assisted fracture fixation or minimally invasive joint preparation for subtalar fusion [4, 6, 10, 12, 13, 16, 17, 18, 19, 23, 24, 26, 27, 30, 33, 34, 36, 40, 46, 52].

Portal placement for arthroscopic approaches to the subtalar joint depends, as in every joint, on the pathology at hand. But one has to keep in mind not only the ailments of the patient, but also the endangered structures surrounding the joints. Complications in arthroscopic procedures around the subtalar joint are reported in up to 6.8% of cases. A frequent cause is connected to the course of neurovascular pathways in the vicinity of the arthroscopic portals around the ankle joints, and, thus, also for subtalar approaches [2, 6, 11, 14, 36, 37, 39, 45]. Nevertheless, these numbers might be over‐ or underestimated due to missing clinical data from larger patient series. Injury to superficial nerves during foot and ankle arthroscopy can be a source of neuropathic pain and chronic pain and can lead, in general, to a poorer functional outcome and lower rate of patient satisfaction [6].

**Figure 1:**
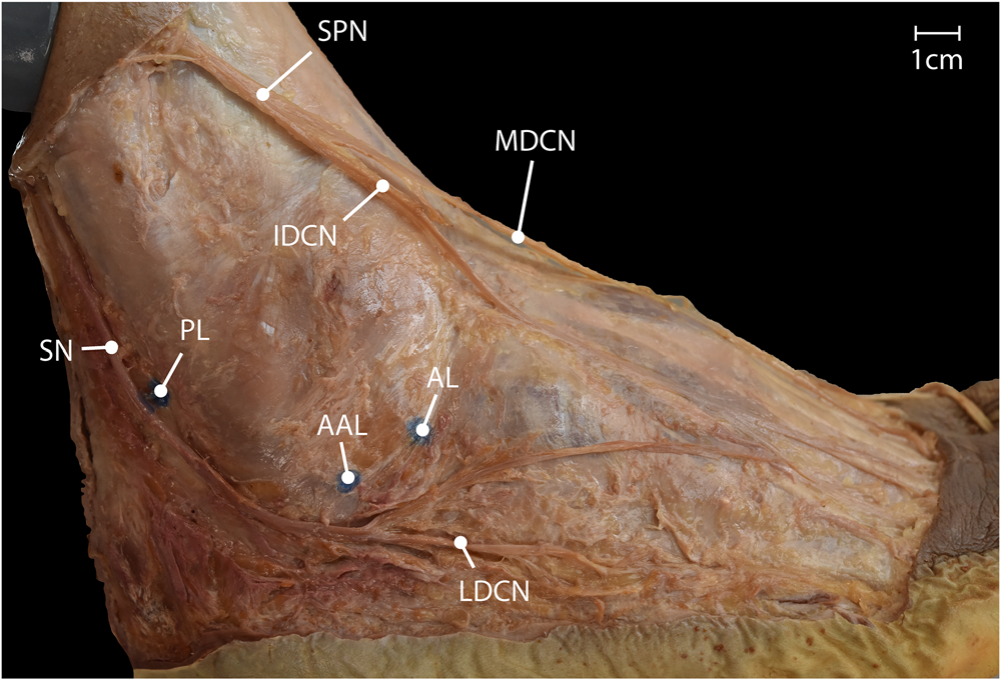
Overview of the subcutaneous nerves on the lateral aspect of the foot and their vicinity to the lateral portals for subtalar arthroscopy. AAL, accessory anterolateral; AL, anterolateral; IDCN, intermediate dorsal cutaneous nerve; LDCN, lateral dorsal cutaneous nerve; MDCN, medial dorsal cutaneous nerve; PL, posterolateral portal; SPN, superficial peroneal nerve; SN, sural nerve. Scale bar = 1 cm.

The skin innervation of the foot and ankle is mainly provided by the saphenous nerve medially, the superficial peroneal nerve (SPN) medio‐dorsally and the sural nerve (SN) postero‐laterally [7, 8]. The SPN subdivides itself into the medial and intermediate dorsal cutaneous nerves. The SN, after being formed in the posterior crural region by the medial sural cutaneous nerve and the peroneal communicating branch, in the majority of cases at approximately 11–20 cm proximal to the lateral malleolus, courses as lateral cutaneous nerve around the lateral malleolus towards the lateral side of the foot (see Figure 1) [15, 35].

Standard arthroscopic portals for a lateral approach to the subtalar joint, as described by Frey et al. [14], are the anterolateral (AL), the middle or accessory anterolateral (AAL) and the posterolateral (PL) portals. Clinical assessment of complication rates regarding iatrogenic nerve injury during standard subtalar arthroscopic portals shows that the AL and AAL portals are associated with low complication rates and the PL portal with an increased risk of SN injury [6, 14, 29, 48, 49].

Current data from anatomic laboratory studies is limited by a low number of included specimens [14, 29, 48], the use of embalmed lower legs [49], and by only simulating arthroscopic portal placement with pin placement [49]. The purpose of this study was to evaluate the distance to surrounding subcutaneous nerves in the vicinity of three standard arthroscopic portals for subtalar joint arthroscopy in a representative number of specimens and through actual portal placement for arthroscopic procedures in order to define anatomical safe zones. The hypothesis of this study was that it is possible to define safe zones for the placement of standard portals for subtalar‐arthroscopy reflecting especially the small safety margin of the PL portal.

## MATERIALS AND METHODS

The study was approved by the ethical committee of the Medical University of Vienna, Austria (EK. Nr. 1524/2020). To evaluate the distance between surrounding subcutaneous nerves and standard portals for subtalar arthroscopy by a lateral approach as described by Frey et al. [14], 40 paired fresh‐frozen foot‐and‐ankle specimens from 20 body donors (thus resulting in 20 right and 20 left specimens) were used. The specimens originated from voluntary and testamentary body donations to the Center for Anatomy and Cell Biology of the Medical University of Vienna, bequeathing their bodies after death for use in teaching and science. For each specimen, age, sex and side were documented.

Specimens were included, if they showed no signs of previous trauma or surgical interventions and no deformity to the region of interest. Exclusion criteria were defined as any signs of moderate to severe osteoarthritis or other pathology influencing the evaluation observed during arthroscopy or subsequent dissection. No specimens had to be excluded during the evaluation.

### Arthroscopy technique

The specimen was placed in a lateral decubitus. The instrument set‐up for subtalar arthroscopy included an 11‐blade, a blunt trocar, a 2.7 mm 30° scope, a small joint shaver 3.0 mm, a chondral pick with 60° curvature, a 22‐gauge needle, and an irrigation pump. A surgical assistant performed subtalar joint inversion and eversion, or ankle dorsiflexion and plantarflexion whenever necessary. No distraction was used. All procedures were performed by the same foot‐and‐ankle‐fellowship‐trained surgeon (M.W.). A three‐portal technique was used as described by Frey et al. [14] using an AL portal, a PL portal and an AAL portal to approach the subtalar joint (see Figure 1). Palpable landmarks, including the lateral malleolus and the Achilles tendon, were marked. The first placed portal was the AL portal about 2 cm anterior to the tip of the fibula at the sinus tarsi. With a 22‐gauge needle, about 8–10 cc of saline was injected into the subtalar joint through the AL portal. The skin was incised vertically using an 11‐blade, followed by the introduction of a mosquito clamp, which was introduced into the sinus tarsi by a nic‐and‐spread technique. Then the blunt trocar was inserted and afterwards switched to the scope. An AAL was created under direct visualization, with an outside‐in technique using a needle to guide portal placement. The AAL portal was posterior to the AL and a bit inferior. Care was taken that the skin bridge between the portals was adequate with at least 1 cm in between portals. After nic and spread with the mosquito clamp, the probe and the shaver were introduced through the AAL working portal. The PL portal was also created under direct visualization, about 1 cm proximal to the tip of the fibula and lateral to the Achilles tendon, in a similar fashion to the AAL portal placement. A systematic point‐by‐point arthroscopic evaluation of the sinus tarsi and the posterior subtalar joint was performed [5, 53]. Wherever needed, a partial synovectomy was performed with the shaver. In order to simulate a micro fracturing procedure at the posterior facet, a chondral pick was inserted through the AAL and PL portals, and three cartilage perforations were created from each portal.

### Dissection

After completion of the subtalar arthroscopy, the arthroscopic portals were marked with pins, the skin was removed in an area between a line approximately 10 cm proximal to the lateral malleolus and the level of the metatarsophalangeal joints. The nerve branches of the SPN or the medial and intermediate dorsal cutaneous nerve and the SN or the lateral dorsal cutaneous nerve were identified and dissected. All nerves were marked with pins upon identification to minimize unintentional movements.

### Evaluation

The specification and origin of the nerves were documented, and the distance of the nerve branches to the AL, AAL and PL portals was measured.

**Figure 2:**
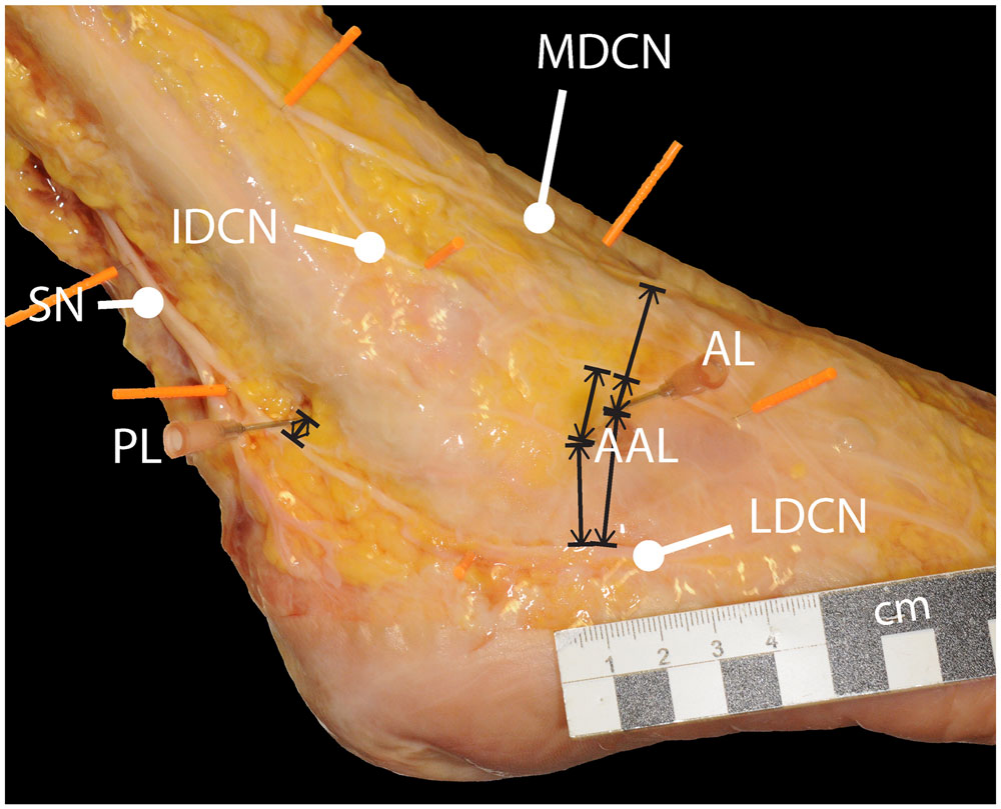
Illustration of the measurements performed. For the AL portal, the distances to the medial, intermediate and lateral dorsal cutaneous nerve were measured. For the AAL portal, the distances to the intermediate and the lateral dorsal cutaneous nerve were measured. For the PL portal, the distance to the sural nerve (SN) was measured. The respective distance of the nerve to the portal was defined as the shortest possible distance. AAL, accessory anterolateral; AL, anterolateral; IDCN, intermediate dorsal cutaneous nerve; LDCN, lateral dorsal cutaneous nerve; MDCN, medial dorsal cutaneous nerve; PL, posterolateral; SPN, superficial peroneal nerve. Scale bar = 1 cm.

Distances between portals and nerves were measured using a digital calliper (accuracy 0.05 mm). For the AL portal, the distances to the medial, intermediate and lateral dorsal cutaneous nerve were measured. For the AAL portal, the distances to the intermediate and the lateral dorsal cutaneous nerve were measured. For the PL portal, the distance to the SN was measured. The respective distance of the nerve to the portal was defined as the shortest possible distance (see Figure 2). All measurements were repeated thrice and in consensus by two investigators (V.B. and M.K.), and the mean was calculated. Every metric variable was documented in mm with two decimals. All dissection steps were documented photographically in a standardized fashion with a commonly available digital camera. Injury to the nerves was evaluated macroscopically and was documented as a ‘verified iatrogenic injury’. Cases of nerve distances <5 mm were counted as ‘possible iatrogenic injury’.

### Statistical analysis

To define the sample size, a sample size calculation was performed during study planning using G*Power [9]. Due to the high variability of measured distances of the subcutaneous nerves in the Refs. [14, 49], a minimum sample size of 16 was required. All data was imputed in a database in SPSS Statistics for Mac Version 27 (IBM Corporation). All metric variables were documented as mean with standard deviation if the data was normally distributed or as median with 25 and 75 percentiles if not. Because all variables followed a Gaussian distribution, metrics were reported as mean with standard deviation with minimum and maximum values. Paired *t* tests were used to check for significant differences regarding the different nerves of the AL and ALL portals and each nearest nerve of the individual portals. A Student's *t* test was used to check for side and sex differences. A *p* < 0.05 was defined as statistically significant. In multiple tests, a Bonferroni correction was applied. The *p* value was therefore corrected for the paired *t* tests to 0.017 for the AL portal and for the comparison between the three portals.

The intraclass correlation coefficient (ICC) for intra‐rater reliability was calculated for the repeated measurements of the nerve distances to the different portals. It ranged between 9.4 and 9.9 reflecting a high repeatability of the measurements. As measurements were performed by two raters in consensus, no ICC for inter‐rater reliability was calculated.

## RESULTS

Forty foot‐and‐ankle specimens were investigated in this study. The mean age of the body donors was 82.9 years (57–101 years); 11 donors were female and 9 were male.

For the AL portal, distances to three cutaneous nerves were measured. It was 28.7 ± 9.2 mm (14.1–58.8 mm) to the medial dorsal cutaneous nerve, 15.4 ± 7.3 mm (1.5–29.0 mm) to the intermediate dorsal cutaneous nerve and 21.7 ± 5.1 mm (12.3–33.41 mm) to the lateral dorsal cutaneous nerve. The distances differed significantly between all nerves (each *p* < 0.001). For the AAL portal, distances to two cutaneous nerves were measured. It was 24.3 ± 6.7 mm (10.3–38.4 mm) to the intermediate dorsal cutaneous nerve and 17.7 ± 4.8 mm (3.6–29.3 mm) to the lateral dorsal cutaneous nerve. The distance differed significantly (*p* < 0.001). For the PL portal, only the distance to the SN was measured, it was 6.7 ± 4.7 mm (0.0–17.6 mm).

**Figure 3:**
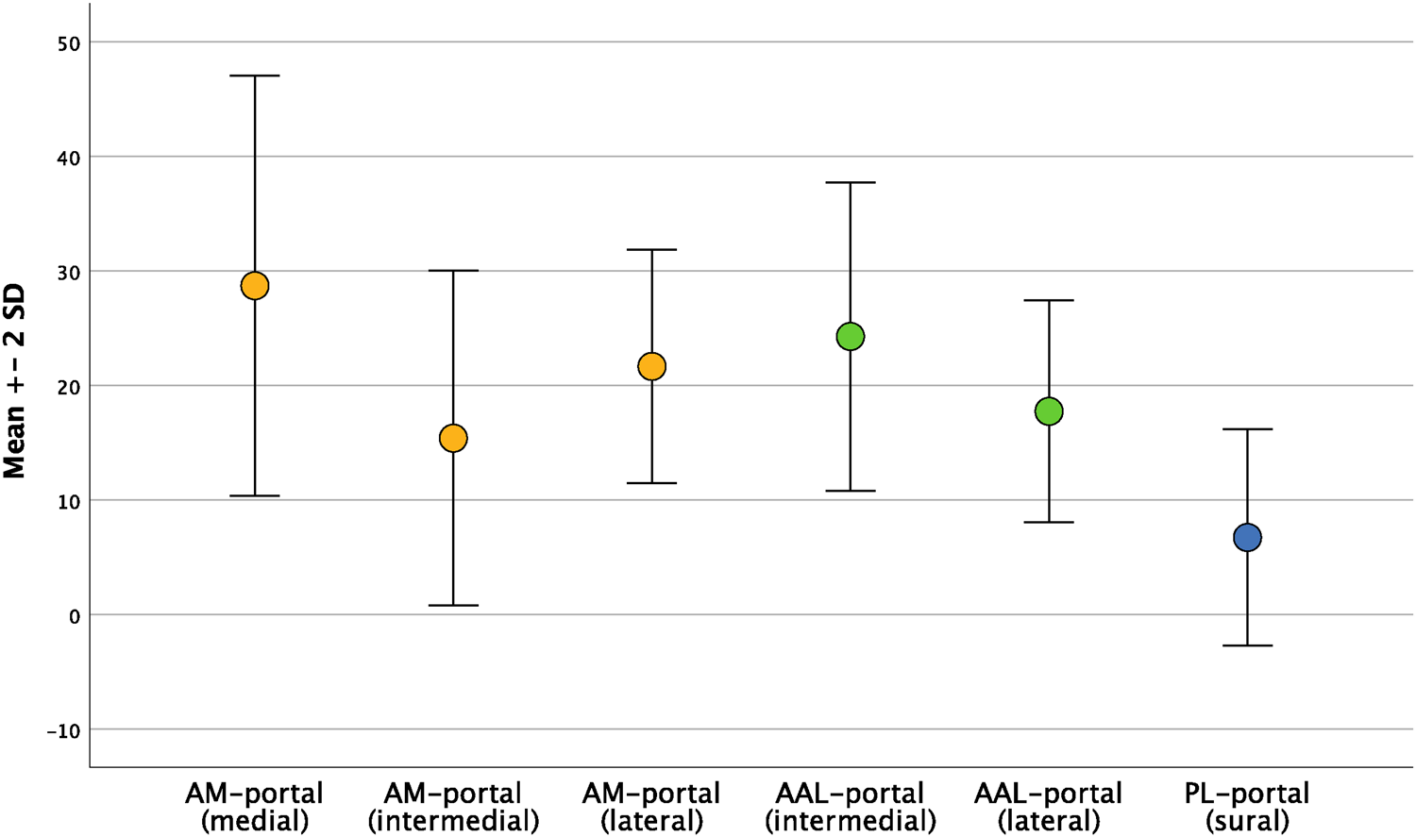
Distribution of distances of the different nerves to the respective portals in mm. Dots signify the mean, whiskers the standard deviation. AAL, accessory anterolateral; AL, anterolateral; IDCN, intermediate dorsal cutaneous nerve; LDCN, lateral dorsal cutaneous nerve; MDCN, medial dorsal cutaneous nerve; PL, posterolateral; SPN, superficial peroneal nerve; SN, sural nerve.

Comparing the distances of the nearest nerve to the respective portal between the three portals, no significant difference was found for the AL and AAL portals (n.s.). The distances differed significantly between the PL portal and the AL portal (*p* < 0.001) as well as the AAL portal (*p* < 0.001) (see Figure 3). In comparing male and female specimens, no statistically significant difference was found (*p* values n.s.). Also comparing left and right specimens, no statistically significant differences were found for any distance between portals or nerves (*p* values n.s.).

During macroscopic evaluation, no case of ‘verified iatrogenic injury’ was found. At the AL portal, two cases (5%) of ‘possible iatrogenic injury’ were found concerning the intermediate dorsal cutaneous nerve. At the AAL portal, one case (2.5%) of ‘possible iatrogenic injury’ was found concerning the lateral dorsal cutaneous nerve. At the PL portal, 16 cases (40%) of ‘possible iatrogenic injury’ were found concerning the SN.

**Figure 4:**
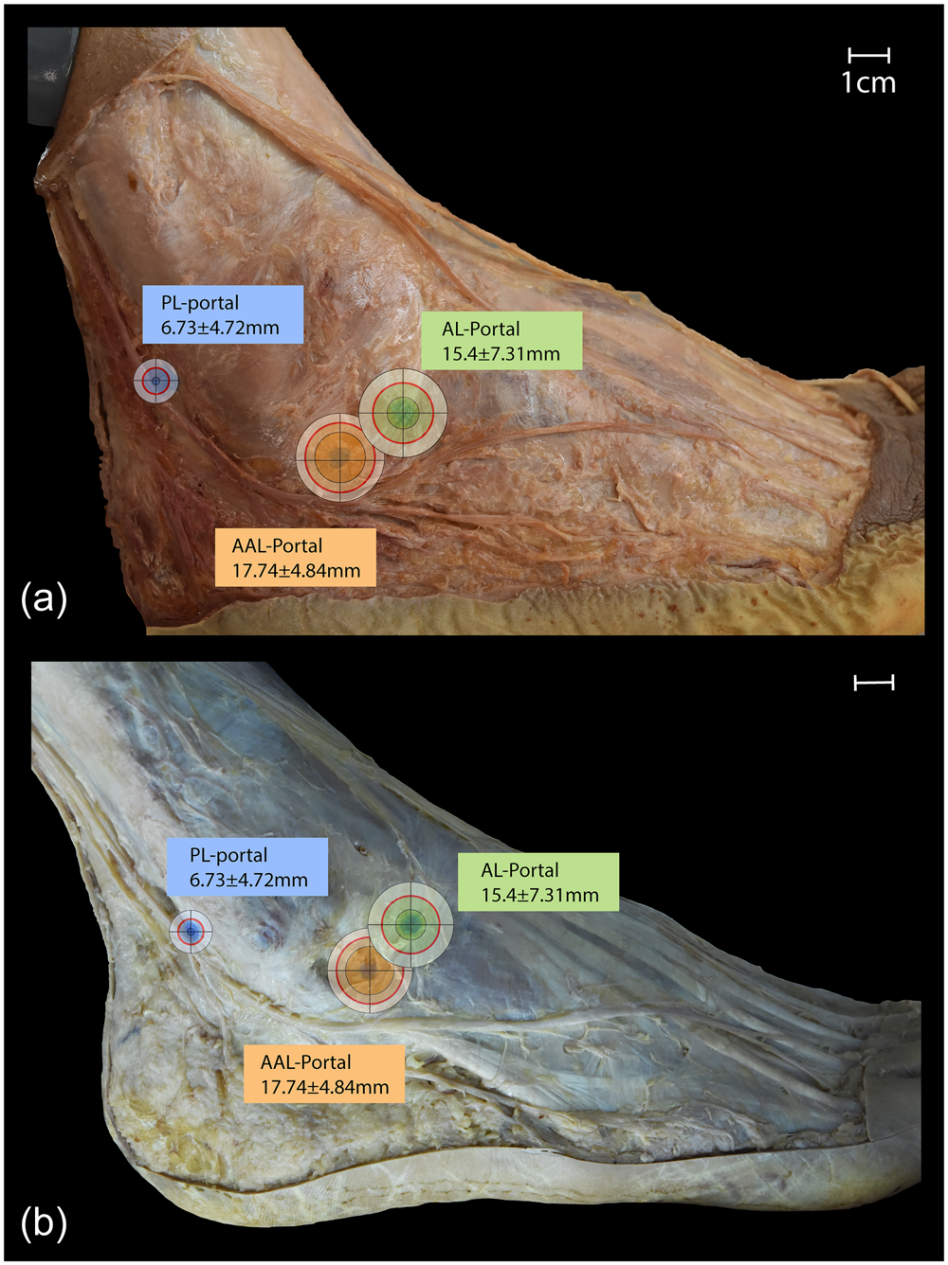
Average safe zones around each portal. Red circle depicts the mean distance between the portal and the nearest nerve. Inner and outer circles show subtracted and added standard deviation. (a) Specimen with superficial peroneal nerve splitting into medial and intermediate dorsal cutaneous nerve and sural nerve continuing into lateral dorsal cutaneous nerve, (b) specimen with superficial peroneal nerve continuing into medial dorsal cutaneous nerve and sural nerve splitting into intermediate and lateral dorsal cutaneous nerve. AAL, accessory anterolateral; AL, anterolateral; PL, posterolateral. Scale bar = 1 cm.

Based on the measured results, safe zones for portal placement were defined (see Figure 4). The portal with the smallest safe zone was the PL portal. The safe zone of the AL and AAL portals is similar in size.

## DISCUSSION

Arthroscopy of the subtalar joint complex is becoming increasingly popular. The major advantages of minimally invasive surgical techniques in foot and ankle surgery, are a lower risk of wound complications, less swelling, and a real‐time three‐dimensional joint evaluation. Safe and effective portal placement is a key element to a successful procedure. It not only enables the best possible access to the joint or the intraarticular pathology, but should also minimize the risks of iatrogenic nerve damage. This study assessed the anatomic course of the superficial nerves at risk during a three‐portal lateral approach subtalar arthroscopy. The SN is the most endangered nerve being 6.7 ± 4.7 mm (0.0–17.6 mm) close to the PL portal. In 16 specimens (40%), the SN sustained a possible injury during arthroscopy.

Iatrogenic nerve injury during surgery is one of the most frequent complications reported after subtalar arthroscopy. Mainly, the SN or its terminal branch, the lateral dorsal cutaneous nerve is reported to be involved, leading to patients complaining of plantar and lateral numbness of the foot. This risk to the SN and the resulting complaints when injured were already frequently reported in the literature for procedures of posterior ankle and hindfoot arthroscopy [14, 20, 21, 28, 31, 47, 50]. In clinical evaluations, SN dysesthesia was reported by Nickisch et al. [31] in seven patients out of a total of 189 performed arthroscopic posterior ankle and hindfoot procedures (3.7%), constituting 44% of all 16 observed post‐operative complications. Abramowitz et al. [1] reported sensory neurapraxia of the SN in 8 (20%) out of 41 patients, and Noguchi et al. [32] in 1 (8%) out of 12 patients. Mouilhade et al. [29] reported SN injury in one (10%) case out of 10 patients. The reported 40% of possible iatrogenic injury to the SN in this study emphasizes the numbers stated in the literature.

The high frequency of nerve injury in arthroscopic approaches to the subtalar joint and in hindfoot arthroscopy, ranging from 3.7% to 20%, led to detailed evaluations of the course of the SN from the ankle region to the lateral foot along with measurements of the distances to the different portals for subtalar arthroscopy.

The SN is formed in the distal third of the lower leg by the combination of the medial sural cutaneous nerve from the tibial nerve and the lateral sural cutaneous nerve from the common peroneal nerve. In its formation, however, there are frequent anatomical variations, possibly also involving a sural communicating branch from the common peroneal nerve. Further distal, it is described to cross the lateral border of the Achilles tendon subcutaneously with a mean of 9.5 cm (6.5–16 cm) proximal to the insertion of the Achilles tendon. From there, the SN courses towards the lateral malleolus, where it runs on average 7 ± 5 mm posterior to the most prominent edge of the lateral malleolus and 13 ± 7 mm distal to its tip. Here, the SN provides highly variable innervation to the malleolar region [20, 44, 51]. Afterwards, it continues as a lateral dorsal cutaneous nerve to the lateral aspect of the foot. Like most subcutaneous nerves, the SN also shows some variability in its course, as was shown, for example, by Duscher et al. [8], and it is also reported to form communicating branches to the SPN, respectively, between the intermediate dorsal cutaneous nerve and the lateral dorsal cutaneous nerve [7].

Also, the SPN shows high variability in its division into the medial and the intermediate dorsal cutaneous nerve as well as in the actual number of branches providing innervation to the dorsal aspect of the foot. After piercing the crural fascia in the distal third of the lower leg, the SPN courses parallel to the tendons of the extensor hallucis longus and extensor digitorum longus towards distal. Similarly to the SN, the SPN provides highly variable branches towards the lateral malleolar region [43, 44]. Due to the course of the SPN, it is less in danger of iatrogenic injury than the SN in subtalar joint arthroscopy. Injury to the SPN or its branches is almost exclusively described for interventions concerning ligament reconstruction on the level of the ankle joint [3, 22, 54].

Looking at the different portals for subtalar joint arthroscopy, the risk for nerve injury is the highest at the level of the PL portal, as here, the SN courses in close proximity of the portal towards distal [14, 29, 49, 51]. In the literature, average posterior–anterior distances range from 4 mm (8–6 mm posterior–anterior) [14] to 11.4 mm (4.4–20.4 mm) on average [49]. This is similar to the findings in this study, reporting 6.7 ± 4.7 mm (0.0–17.6 mm) to the PL portal. Here, one has to emphasize, that also distances of 0.00 mm to the PL portal were measured, which is in contrast to previous studies, although Frey et al. [14] reported one case of SN transection. This was also the reason, why a cut‐off of 5 mm was defined as relevant for identifying possible iatrogenic nerve injury in this study. As a result, the close proximity of the SN of the PL portal has to be taken into account when performing subtalar arthroscopic procedures. In order to avoid possible nerve damage, the surgeon has to be thoughtful with the surgical technique of portal placement (i.e., nick and spread technique) and careful during the introduction of sharp instruments. Patients have to be consented for possible hypoesthesia, dysaesthesia or neuropathy at the sensory area of the SN, or even neuroma formation around the PL portal [38, 41, 42].

Instead of the often cited middle portal, the AAL portal was used for evaluation. Distances measured 24.3 ± 6.7 mm (10.3–38.4 mm) to the intermediate dorsal cutaneous nerve and 17.7 ± 4.8 mm (3.6–29.3 mm) to the lateral dorsal cutaneous nerve. These distances, however, approximately match also reports for the middle portal, measuring a distance of 20.9 mm (5.8–37.6 mm) to the lateral dorsal cutaneous nerve [49]. The middle portal was also reported as showing the least risk of nerve injury [14], which is also supported by the results of this study.

The AL portal has three subcutaneous nerve branches in its vicinity. The distance to the medial dorsal cutaneous nerve was 28.7 ± 9.2 mm (14.1–58.8 mm), 15.4 ± 7.3 mm (1.5–29.0 mm) to the intermediate dorsal cutaneous nerve and 21.7 ± 5.1 mm (12.3–33.4 mm) to the lateral dorsal cutaneous nerve. Lintz et al. [25] described the distance from the AL portal to the lowest branch of the SPN, that is, the intermediate dorsal cutaneous nerve, as 16.7 ± 8.4 mm with a minimum of 4 mm and to the highest branches of the SN, that is, the lateral dorsal cutaneous nerve as 12.2 ± 5.3 mm also with a minimum of 4 mm. The distance to the intermediate dorsal cutaneous nerve is similar in this study, but the distance to the lateral dorsal cutaneous nerve is significantly smaller, as reported in the results. Also, Frey et al. [14] reported similar distances for the intermediate dorsal cutaneous nerve (17 mm, range 0–28 mm) but significantly smaller distances for the lateral dorsal cutaneous nerve (8 mm, range 2–12 mm). On the other hand, Tryfonidis et al. [49] reported similar distances to the lateral dorsal cutaneous nerve (21.3 mm, range 0–35.5 mm).

The results of this study are in accordance with the available literature, which shows an unequal risk distribution for nerve injuries among available portals for subtalar joint arthroscopy [49]. Damage to the surrounding subcutaneous nerves is significantly less likely in AL, AAL and middle portals and significantly more likely in the PL portal. The authors therefore recommend the placement of a PL portal only in cases, when the posterior subtalar joint has to be assessed or addressed during surgery. Apart from the possibility of protecting subcutaneous nerves during surgery through identification and retraction by blunt dissection, through the meticulous use of a nick‐and‐spread technique [1, 32], exact anatomical guidance is needed to decrease the incidence of iatrogenic nerve injury during surgery. By defining anatomical safe zones and reporting the rates of iatrogenic nerve injury for each portal, surgeons receive important information for accurate portal placement.

## LIMITATIONS

There are some limitations to acknowledge when considering the results of this study. First of all, the number of specimens is not adequate to reflect the high variability of the course of subcutaneous nerves. This is, therefore, the reason why the individual variants of subcutaneous nerves of the dorsum of the foot were not included in the aims of this study and the exact branching pattern of each specimen was not specifically evaluated. Anatomical variations of the subcutaneous nerves were, while observed during dissection, not evaluated in this study. Although it is, of course, important also from a clinical point of view to know the most common nerve branches and variations, it would, in the daily routine, not be feasible to assess each patient regarding his or her anatomical variants. The presented safe zones per se are defined by a large collective of human specimens, which nevertheless do not guarantee 100% safety to avoid nerve injury. Additionally, fresh‐frozen specimens were used, in which slippage of soft tissue structures may occur unintentionally, contrary to embalmed specimens. To minimize this, all subcutaneous nerves were fixed with pins to the underlying tissue. Also, all measurements—although repeated—were performed in consensus by two raters and not individually. Thus, no inter‐rater reliability was calculated. However, as the intra‐rater reliability showed a high agreement, and the measurements were performed in consensus by two raters, possible bias should be minimized.

## CONCLUSION

Placement of the AL and AAL portals in subtalar arthroscopy is safe using standard anatomical landmarks and a thorough surgical technique. The nearest subcutaneous nerve branch of the SPN was at least 1 cm distant from the AL and AAL portals. Surgeons should be aware that at the level of the PL portal, the SN is the most endangered structure in subtalar arthroscopy, and, thus, take the utmost care during portal placement and instrument insertion to avoid iatrogenic injury.

## AUTHOR CONTRIBUTIONS

*Conzeptialization*: Lena Hirtler and Madeleine Willegger. *Data curation*: all authors. *Formal analysis*: Lena Hirtler. *Investigation*: all authors. *Methodology*: Lena Hirtler and Madeleine Willegger. *Project administration*: Lena Hirtler. *Recources*: Lena Hirtler and Madeleine Willegger. *Supervision*: Lena Hirtler and Madeleine Willegger. *Validation*: Lena Hirtler. *Visualization*: Lena Hirtler. *Writing—original draft preparation*: Lena Hirtler. Writing—review and editing: all authors.

## CONFLICT OF INTEREST STATEMENT

The authors declare no conflict of interest.

## ETHICS STATEMENT

Ethical approval for the study was obtained from the Ethics Committee of the Medical University of Vienna (EK. Nr. 1520/2020).

## Data Availability

Data that support the findings of this study are available from the corresponding author upon reasonable request.
